# Miscellaneous

**Published:** 1890-03

**Authors:** 


### MISCELLANEOUS.

Colored Spectacles.—Dr. Konigstein, while giving directions in his class of the uses and prescribing of spectacles, said that green glass as a protection against strong rays was worse than useless, and did more harm to a sensiiive eye than good, as they allowed the yellow rays to be transmitted and unnecessarily irritate the eye. As a protection against strong rays the blue or smoked glasses were the only protection.

Paper Pillows.—The latest fad in England is paper pillows. The paper is torn into very small pieces, not bigger than the finger nail, and then put into a pillow sack of drilling or light ticking. They are very cool for hot climates, and much superior to feather pillows. The newspapers are printing appeals for them for hospitals. Newspapers are not nice to use, as there is a disagreeable odor from printers' ink; but brown or white paper and old letters and envelopes are the best. As they are torn, stuff them into an old pillow-case, and it can be seen when there are enough. The easiest way is to tear or cut the paper in strips about half an inch wide, and then tear or cut across. The finer it is, the lighter it makes the pillows.

Prof. Loisette's Memory System is creating greater interest than ever in all parts of the country, and persons wishing to improve their memory should send for his prospectus, free as advertised in another column.

Facts to Remember.—A cubit is two feet. A space is three feet. A fathom is six feet. A span is 10½ inches. A palm is three inches. A league is three miles. A great cubit is 11 feet. Two persons die every second. Sound moves 743 miles per hour. A square mile contains 640 acres. An acre contains 4,840 square yards. A hand (horse measure) is four inches.

Japanese Lacquer is the product of a tree, the Rhus vernicifera, D. C., which grows throughout the main island of Japan. It attains a large size, the trunks sometimes measuring a metre in diameter. It is said the tree will live for forty years, but only comparatively young trees are valued for the production of lacquer. Haviug yielded for several years they are cut down, the lacquer extracted from the branches, and young trees take their places. "It gives a surface to wood," Mr. Hitchcock says, "much harder than our best copal varnish, without brittleness. It takes a polish not to be excelled, which lasts for centuries, as we may see in the old treasures of Japan. It is proof against boiling wator, alcohol, and indeed, it seems to be insoluble in every agent known. It is the best possible application for laboratory tables. I have a set of photographer's developing trays that have been in use for more than a year, and I find them excellent and cheap. In Japan it is used for many household articles." Unfortunately, lacquer poisoning from the fresh material is a serious danger. According to Rein, the poison is a volatile acid, and Mr. Hitchcock suggests that it might be removed by a heat that would leave the lacquer uninjured.

### A CAPTURE.

"There's magic in your glance,
  Fair maid,
A witchery to entrance,"
  He said.
"I own the fateful charm,
I yield me, I disarm,"
  He said.

"No knight I captive take
  Self-made.
You must your ransom make,"
  She said.
"Besides, what use my charms
If you've laid down your arms,"
  She said.

—Loring G. Bany.

